# Correlation of non-esterified fatty acids with acute coronary syndrome risk in young Chinese adults

**DOI:** 10.3389/fendo.2025.1479497

**Published:** 2025-06-06

**Authors:** Jian-di Wu, Jian-jing Luo, Jia-huan Li, Sha-li Hao, Wen-li Wang, Wu Li, Wei-wen Li, Guo-lin Huang, Guo-quan Liang, Wei-xing Wen, Wei-min He, Yang-guang Liu, Yang-xin Chen, Xiao-mei Zhang, Zao-peng He, Yuli Huang

**Affiliations:** ^1^ Department of Cardiology, The Second People’s Hospital of Foshan, Foshan, Guangdong, China; ^2^ Department of Cardiology, The Eighth Affiliated Hospital, Southern Medical University (The First People’s Hospital of Shunde Foshan), Foshan, China; ^3^ Department of Internal Medicine, Zhaoqing Medical Collage, Zhaoqing, China; ^4^ Department of Cardiology, The Affiliated Chencun Hospital of Shunde Hospital, Southern Medical University, Foshan, China; ^5^ Department of Cardiology, The Second Hospital of Zhaoqing, Zhaoqing, Guangdong, China; ^6^ Department of Cardiology, The Sixth Affiliated Hospital of South China University of Technology, Foshan, Guangdong, China; ^7^ Department of Cardiology, Sun Yat-sen Memorial Hospital, Sun Yat-sen University, Guangzhou, China; ^8^ Department of Cardiology, Lecong Hospital of Shunde, Guangdong, Foshan, China; ^9^ Guangdong Provincial Key Laboratory of Cardiac Function and Microcirculation, Guangzhou, China

**Keywords:** non-esterified fatty acids, free fatty acids, risk factors, coronary artery disease, acute coronary syndrome, dyslipidemia

## Abstract

**Objective:**

Circulating non-esterified fatty acids (NEFAs) are linked to endothelial dysfunction and coronary artery disease (CAD) mainly in older adults. This study examines the association between NEFAs and acute coronary syndrome (ACS) risk in young Chinese individuals.

**Method:**

Of the 1264 young ACS patients and 1072 age-matched controls aged 55 years or younger assessed, 1108 ACS patients and 979 controls were found eligible. Their conventional cardiovascular risk factors were compared, and serum NEFA levels were determined using a commercial assay kit.

**Result:**

ACS patients exhibited a higher prevalence of male sex, smoking, hypertension, diabetes and overweight/obesity compared to controls. Additionally, ACS patients had elevated levels of low-density lipoprotein cholesterol, triglycerides, and NEFAs, along with reduced levels of estimated glomerular filtration rate and high-density lipoprotein cholesterol (all *P* < 0.05). After adjusting for various cardiovascular risk factors through multivariate logistic regression, NEFA levels remained independently associated with ACS risk in young patients (per 100 µmol/L increase, OR = 1.207, 95% CI = 1.163–1.253). Restricted cubic spline analysis confirmed a linear relationship between NEFA levels and ACS risk. Furthermore, receiver operating characteristic analysis indicated that NEFA levels have significant diagnostic value for ACS in young patients, with an area under the curve of 0.62 (*P* < 0.001).

**Conclusion:**

Elevated circulating NEFA levels could be linked with increased ACS risks in young Chinese individuals, regardless of traditional cardiovascular risk factors.

## 1 Background

Cardiovascular disease (CVD) is the leading cause of death worldwide. Although traditionally observed in the elderly, recent epidemiological data indicate a rising incidence among younger individuals, particularly in coronary artery disease (CAD) (1). Young and middle-aged individuals are more likely to experience acute coronary syndrome (ACS) rather than stable CAD, with approximately 25% of ACS patients aged <55 years (2), representing a substantial burden on public health systems. Thus, early identification and management of ACS risk factors in young adults are crucial for reducing the global burden of CVD.

Traditional cardiovascular (CV) risk factors (i.e., high blood pressure, dyslipidemia, and diabetes are less common in younger individuals compared to older individuals. This gap highlights the need to identify novel risk factors for premature CVD. Our previous research has highlighted several potential biomarkers linked to lipid metabolism, oxidative stress, metabolic dysfunction, immune activation, and low-grade inflammation as risk factors in younger and middle-aged CAD patients (3–6).

Non-esterified fatty acids (NEFAs), also known as free fatty acids (FFAs), are primarily produced through the hydrolysis of fats (lipolysis) and can also be synthesized in organs such as the mammary glands, liver, and adipose tissue (7). Under normal conditions, NEFAs act as energy substrates, particularly during fasting. However, elevated NEFA levels have been associated with adverse health outcomes, including diabetes mellitus (DM), CVD and heart failure (8–11), and these associations are thought to involve chronic inflammation and endothelial dysfunction (12, 13). Cardiovascular events in young patients often occur in the absence of extensive atherosclerosis but with prominent inflammatory components, understanding NEFA’s pro-inflammatory role becomes particularly relevant. However, until now, most of the related studies have focused on elderly populations.

In this present study, we aimed to evaluate the association between circulating NEFA levels and ACS risks in a young Chinese cohort and investigate how traditional CV risk factors relate to NEFAs in this cohort.

## 2 Method

### 2.1 Ethics and study eligibility

This retrospective study was conducted in accordance with the Declaration of Helsinki and received approval from the Ethics Committee of Shunde Hospital, Southern Medical University, China (No: KY20191103). The participants provided signed consent.

As previously reported (1, 3–5), the inclusion criteria for young patients with ACS were: (1) individuals aged 55 years or younger; (2) presented with acute myocardial ischemia within the recent 2 months; (3) exhibited stenosis of ≥50% in at least one major coronary artery, as determined by coronary angiography (CAG). The cases were retrieved from the participating institutions. CAG was conducted via the radial artery using the Judkins technique, with the femoral artery as an alternative if needed. Results were assessed by two interventional cardiologists and reviewed by a radiologist.

Exclusion criteria including: (1) suspected acute myocarditis or stress cardiomyopathy; (2) myocardial infarction with non-obstructive coronary arteries (MINOCA), due to its different pathophysiology compared to obstructive coronary conditions; (3) uncontrolled infectious or autoimmune diseases, severe renal dysfunction (eGFR < 30 mL/min/1.73 m²) or renal replacement therapy, acute hepatitis, mental disorders, type 1 DM, or cancer; and (4) used hormone replacement therapy, fish oil or polyunsaturated fatty acid supplements in the recent 3 months, as these interventions may interfere with endogenous FFA metabolism or introduce confounding factors, such as anti - inflammatory and lipid - regulating effects.

Control subjects comprised age-matched individuals with negative results from either CAG or coronary computed tomography angiography (CTA). Clinicians chose between coronary CTA and CAG based on the patient’s chest discomfort and signs of myocardial ischemia (i.e., ST-segment deviations on the ECG, positive treadmill tests, regional dyskinesia on echocardiography), or diagnostic uncertainties.

### 2.2 Study indicators

Venous blood samples were taken after ≥8 hours of fasting and assessed for fasting blood glucose (FBG), glycated hemoglobin (HbA1c), total cholesterol (TC), high-density lipoprotein cholesterol (HDL-C), triglycerides (TG), low-density lipoprotein cholesterol (LDL-C), high-sensitivity C-reactive protein (hs-CRP), and serum creatinine (Scr). The corresponding results were retrieved from medical records for analysis.

The serum samples were preserved at -80°C until NEFA analysis. Briefly, total serum NEFA levels were measured using a commercial NEFA assay kit (ACS-ACOD method) from Beijing Strong Biotechnologies (Beijing, China) with an automatic biochemistry analyzer (Beckman AU5800, California, USA). This method quantifies the collective concentration of all non-esterified fatty acids (including both long-chain and short-chain species) via enzymatic colorimetric detection of free carboxyl groups. All measurements were performed according to the manufacturer’s instructions. All measurements were performed according to the manufacturer’s instructions.

### 2.3 Covariates for ACS

Conventional ACS risk factors were defined as follows: A family history of premature CAD included CAD in a first-degree male relative under 55 or a female relative under 65. Smokers were those who had smoked regularly in the past year, while non-smokers had never smoked or had quit for over a year. Hypertension was defined as a systolic blood pressure ≥140 mmHg and/or diastolic blood pressure ≥90 mmHg or current antihypertensive use (14). DM was defined as fasting blood glucose ≥7.0 mmol/L, HbA1c ≥6.5%, or hypoglycemic treatment (15). Dyslipidemia included total cholesterol ≥5.18 mmol/L, LDL-C ≥3.37 mmol/L, HDL-C <1.04 mmol/L, TG ≥1.7 mmol/L, or anti-dyslipidemia treatment (16). Overweight was a BMI of 24.0-27.9 kg/m², and obesity was a BMI ≥28 kg/m², as per Chinese criteria (17). Renal function was evaluated by eGFR using the modified MDRD equation for Chinese individuals (18).

### 2.4 Statistical assessments

Statistical analyses were performed with SPSS Statistics (Windows v23.0, IBM Corp., Armonk, NY, USA). Continuous variables are presented as mean ± standard deviation (SD) for normally distributed data or median with interquartile range (IQR) for non-normally distributed data. Categorical variables were expressed as numbers and proportions. ACS patients and controls were compared using the Wilcoxon rank-sum test for non-normally distributed variables, the two-tailed *t*-test for normally distributed variables, and the chi-square or Fisher’s exact test for categorical variables.

To handle missing data (<5%), we used multivariate imputation by chained equations with 10 imputations. This method was selected for its capacity to model complex missing-at-random mechanisms, preserve multivariate relationships through fully conditional specification, and generate robust estimates by accounting for nonlinear interactions among variables. Correlations between covariates and NEFAs were evaluated using Pearson’s correlation coefficient. Variables with non-Gaussian distributions were logarithmically transformed. Univariate and multivariate logistic regression analyses identified factors linked to ACS. Covariates included sex, age, smoking status, CAD family history, BMI, diabetes, hypertension, lipid profiles, eGFR, hs-CRP, and NEFAs. Covariates with P <0.10 in univariate analysis were included in multivariate regression. Adjusted odds ratios (ORs) and 95% confidence intervals (CIs) were computed.

To assess the dose-response relationship between NEFAs and ACS risk, a four-node restricted cubic spline (RCS) was used, including the 5th, 35th, 65th, and 95th percentiles. Receiver operating characteristic (ROC) curve analysis was conducted, and the area under the curve (AUC) was calculated to determine NEFAs’ diagnostic value for ACS in young patients. Statistical significance was set at *P* < 0.05.

## 3 Results

### 3.1 Baseline characteristics

**Figure 1:**
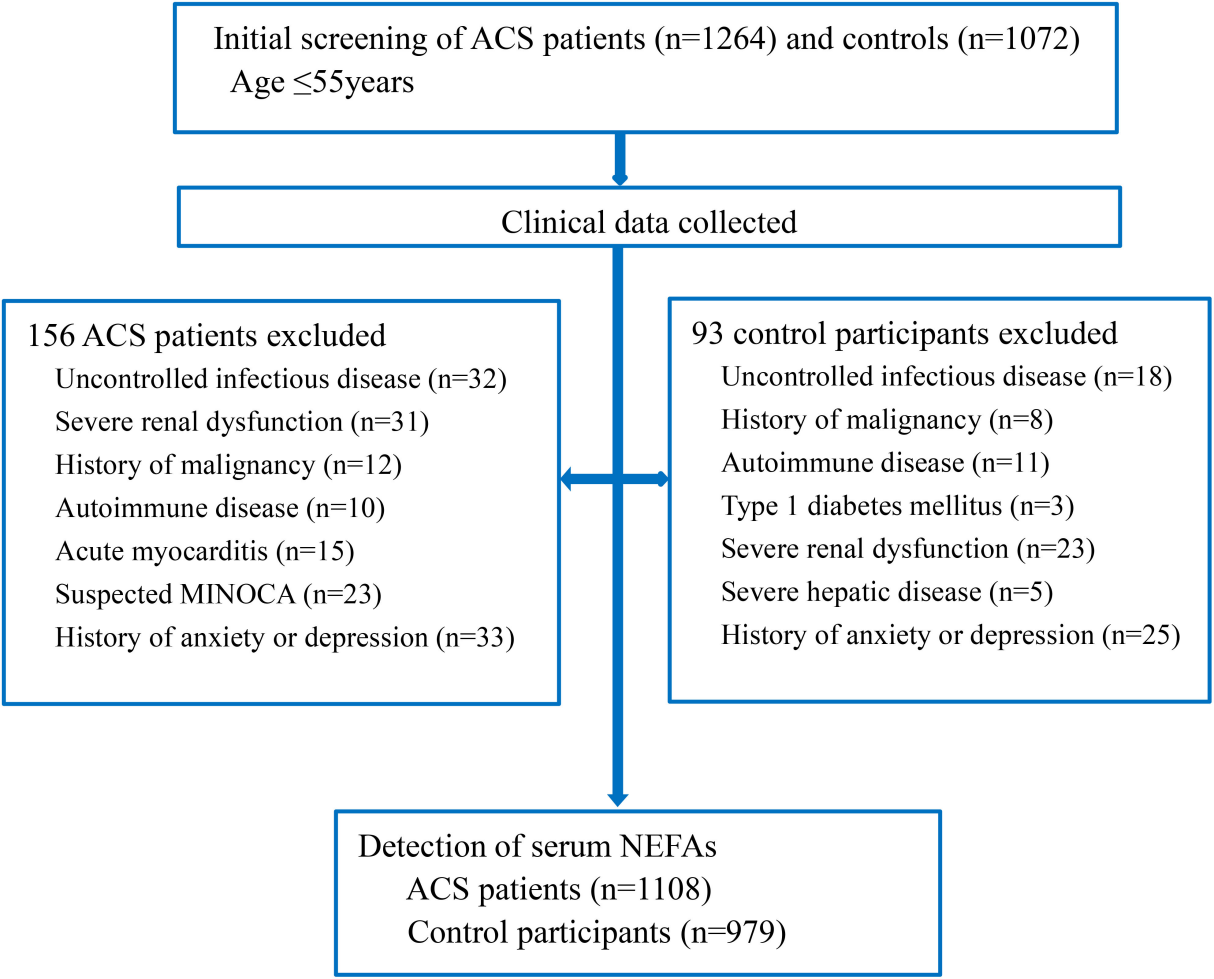
Flow chart of the study. ACS, acute coronary syndrome; MINOCA, myocardial infarction and non-obstructive coronary; NEFAs, non-esterified fatty acids.

We reviewed 1,264 young ACS patients and 1,072 age-matched controls (all ≤55 years old). After applying exclusion criteria, 156 ACS patients and 93 controls were removed. The final cohort included 1,108 ACS patients (897 men, 211 women) and 979 controls (460 men, 519 women) (**Figure 1**). Of the ACS patients, 346 had unstable angina, 405 had non-ST-segment elevation myocardial infarction, and 357 had ST-segment elevation myocardial infarction. Demographic and clinical characteristics are provided in **Table 1**.

**Table 1:** Demographic and clinical characteristics of CAD patients and controls.

Variables	All participants (n=2087)	CAD group (n=1108)	Control group (n=979)	*P* value
Age (years)	48.4 ± 5.6	48.5 ± 4.7	48.2 ± 6.4	0.194
Men [n(%)]	1357 (65.0%)	897 (81.0%)	460 (47.0%)	<0.001
CAD family history [n(%)]	84 (4.0%)	56 (5.1%)	28 (2.9%)	0.01
Current smokers [n(%)]	749 (35.9%)	468 (42.2%)	281 (28.7%)	<0.001
Hypertension [n(%)]	722 (34.6%)	421 (38.0%)	301 (30.7%)	<0.001
SBP (mm Hg)	132.1 ± 20.8	132.2 ± 21.0	131.1 ± 20.6	0.925
DBP (mm Hg)	81.2 ± 12.7	81.7 ± 12.3	80.8 ± 14.2	0.126
DM [n(%)]	549 (26.3%)	313 (28.2%)	236 (24.1%)	0.032
FBG (mmol/L)	7.34 ± 3.76	7.41 ± 3.58	7.26 ± 3.96	0.349
HbA1c	6.21 ± 1.36	6.22 ± 1.23	6.20 ± 1.50	0.758
TC (mmol/L)	4.32 (3.71-5.25)	4.31 (3.70-5.27)	4.32 (3.78-5.22)	0.119
LDL-C (mmol/L)	2.52 (2.02-3.14)	2.58 (1.97-3.29)	2.50 (2.03-2.90)	<0.001
HDL-C (mmol/L)	1.05 (0.87-1.23)	1.00(0.85-1.20)	1.10(0.89-1.29)	<0.001
TG (mmol/L)	1.49 (1.05-2.23)	1.68 (1.17-2.47)	1.30 (0.98-1.88)	<0.001
Overweight/Obesity [n(%)]	544 (26.1%)	324 (29.2%)	220 (22.5%)	<0.001
BMI (kg/m^2^)	24.9 ± 4.8	24.9 ± 4.8	24.9 ± 4.8	0.891
HR (beats/minute)	76.2 ± 16.6	76.9 ± 16.3	75.3 ± 16.9	0.028
eGFR (mL/min/1.73 m2)	88.9 ± 21.0	87.0 ± 20.3	90.9 ± 21.5	<0.001
hs-CRP (mg/L)	4.20 (1.67-8.20)	4.30 (2.00-8.11)	4.10 (1.56-8.29)	0.157
NEFA (μmol/L)	723.9 ± 286.9	782.3 ± 292.0	657.8 ± 266.1	<0.001

Data are presented as percentages, mean and SD, median and interquartile range.

BMI, Body mass index; CAD, coronary artery disease; DBP, diastolic blood pressure; DM, diabetes mellitus; eGFR, estimated glomerular filtration rate; FBG, fasting blood glucose; HDL-C, High density lipoprotein-cholesterol; HR, heart rate; hs-CRP, high-sensitivity C-reactive protein; LDL-C, Low density lipoprotein-cholesterol; NEFA: non-esterified fatty acids; SBP, systolic blood pressure; TC, Total cholesterol; TG, Triglyceride.

As presented in **Table 1**, ACS patients comprised more men, smokers and individuals with hypertension, diabetes, and overweight/obesity compared to controls, and also had higher LDL-C, TG and NEFAs levels, and lower eGFR and HDL-C levels (all *P* < 0.05). Other cardiovascular risk factors did not differ significantly between the groups.

### 3.2 Impact of serum NEFAs and other CV risk factors

**Table 2** presents the correlations between serum NEFAs and clinical variables. NEFAs levels were positively correlated with age, HbA1c, TC, LDL-C, TG and hs-CRP, negatively correlated with eGFR (all *P* < 0.05), and no other significant correlations were found.

**Table 2:** Correlation of NEFA and other cardiovascular risk factors.

Variables	r value	P value
Age (years)	0.061	0.005
Sex (female)	0.003	0.880
Smoking (yes)	0.010	0.648
SBP (mm Hg)	0.011	0.609
DBP (mm Hg)	0.007	0.753
FPG (mmol/L)	-0.031	0.153
HbA1C (%)	0.045	0.041
Ln(TC) (mmol/L)	0.054	0.014
Ln (LDL-C) (mmol/L)	0.098	<0.001
Ln (HDL-C) (mmol/L)	-0.024	0.277
Ln (TG) (mmol/L)	0.102	<0.001
BMI (kg/m^2^)	-0.002	0.913
eGFR (mL/min/1.73 m2)	-0.059	0.007
Ln (hs-CRP) (mg/L)	0.121	<0.001

TC, HDL-C, LDL-C, TG and hs-CRP were skewed variables and logarithmically transformed.

BMI, Body mass index; DBP, diastolic blood pressure; eGFR, estimated glomerular filtration rate; FBG, fasting blood glucose; HDL-C, High density lipoprotein-cholesterol; HR, heart rate; hs-CRP, high-sensitivity C-reactive protein; LDL-C, Low density lipoprotein-cholesterol; NEFA, non-esterified fatty acids; SBP, systolic blood pressure; TC, Total cholesterol; TG, Triglyceride.

### 3.3 Serum NEFAs and ACS risks in young cases

Our univariate logistic regression found that male sex, smoking, family history of CAD, diabetes, hypertension, and higher levels of TG, TC, LDL-C, hs-CRP, and NEFAs were linked to increased ACS risk in young patients. Elevated HDL-C and eGFR levels were associated with reduced ACS risk (**Table 3**).

**Table 3:** Risk factors for CAD in young patients in univariate logistic regression analysis.

Risk factors	OR	95% CI	P-value
Sex (male vs female)	4.796	3.945-5.831	<0.001
Age (per 10 years)	1.107	0.950-1.292	0.194
Smoking (yes vs no)	1.816	1.513-2.181	<0.001
CAD family history (yes *vs* no)	1.808	1.139-2.870	0.01
BMI (per 1.0 kg/m^2^)	1.001	0.983-1.019	0.891
DM (yes *vs* no)	1.240	1.019-1.508	0.032
Hypertension (yes *vs* no)	1.380	1.151-1.656	<0.001
TG (per 1 mmol/L)	1.290	1.20-1.387	<0.001
TC (per 1 mmol/L)	1.117	1.042-1.196	0.002
LDL-C (per 1 mmol/L)	1.301	1.181-1.433	<0.001
HDL-C (per 1 mmol/L)	0.43	0.311-0.594	<0.001
eGFR (per 10 mL/min/1.73 m^2^)	0.914	0.877-0.953	<0.001
hs-CRP (per 1 mg/ml)	1.015	1.001-1.030	0.038
NEFA (per 100μmol/L)	1.173	1.136-1.211	<0.001

BMI, body mass index; CAD, coronary artery disease; DM, diabetes mellitus; CI, confidence interval; eGFR, estimated glomerular filtration rate; HDL-C, high-density lipoprotein cholesterol; hs-CRP, high-sensitivity C-reactive protein; LDL-C, low-density lipoprotein cholesterol; OR, odds ratio; NEFA, non-esterified fatty acids; TC, total cholesterol; TG, triglyceride.

**Figure 2:**
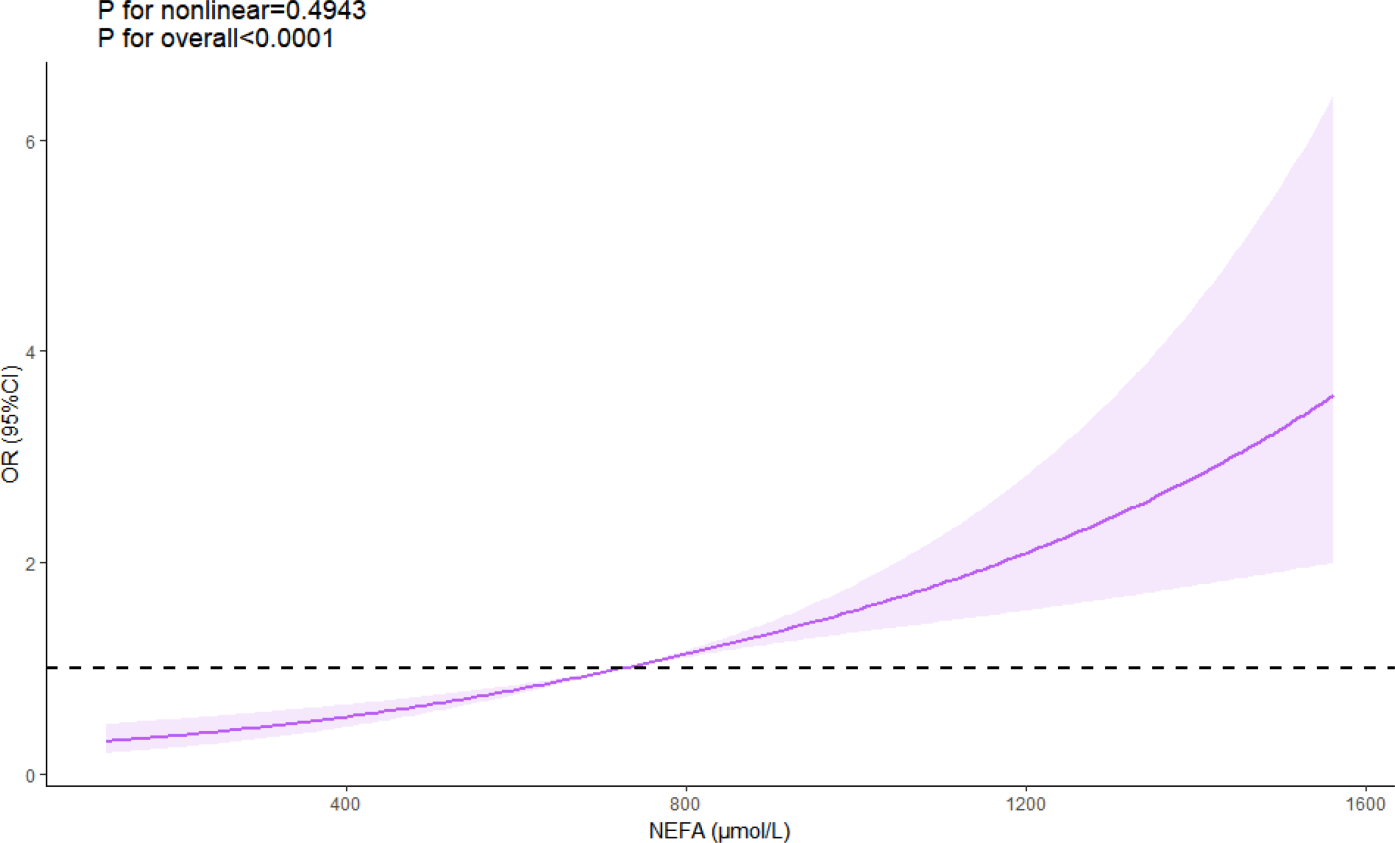
Linear relationship between NEFA levels and ACS risk. ACS, acute coronary syndrome; NEFAs, non-esterified fatty acids.

**Figure 3:**
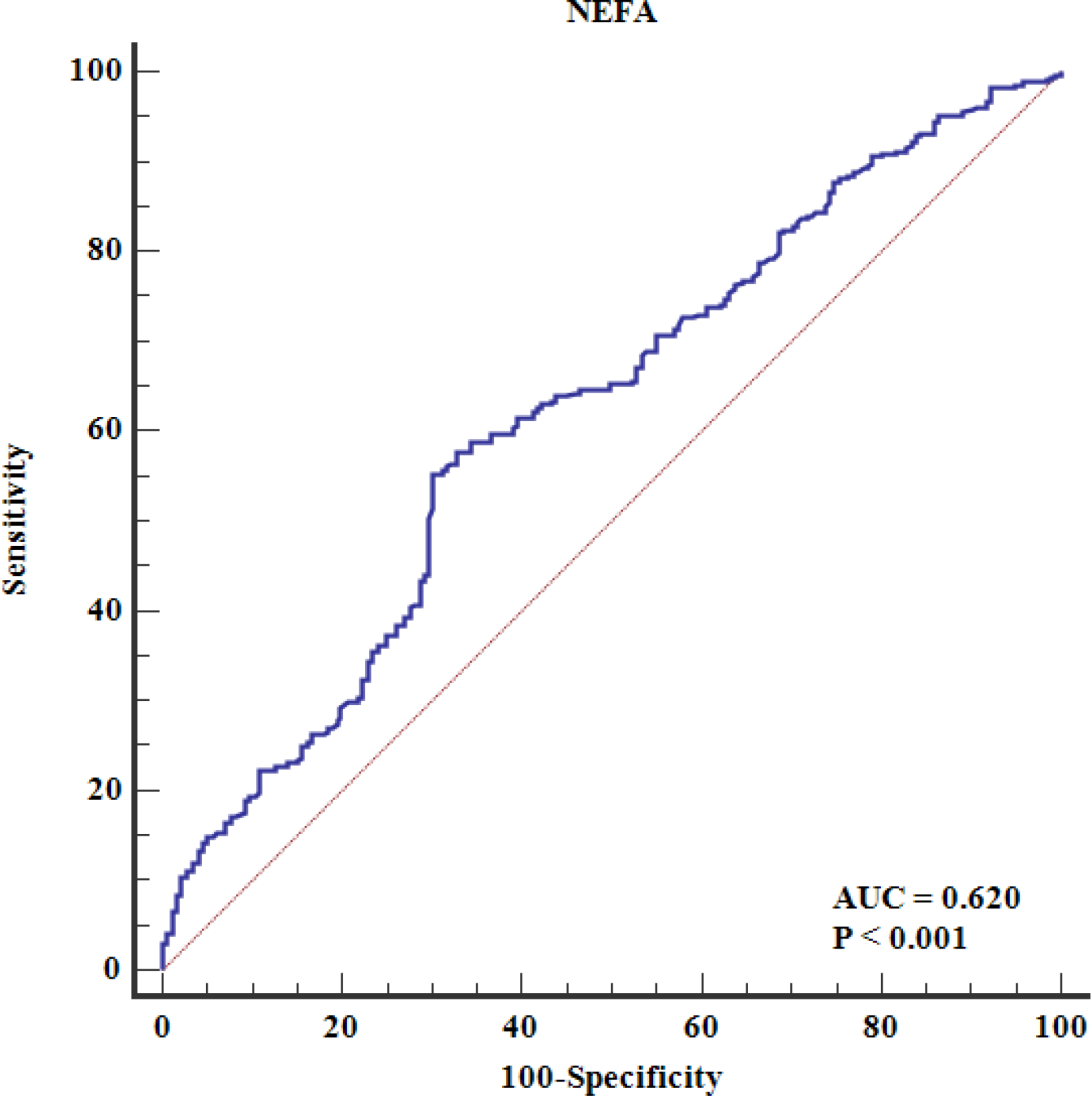
The ROC curve analysis of serum NEFAs in predicting premature CAD. The ROC analysis evaluates the diagnostic performance of NEFAs alone (unadjusted model). The area under the curve (AUC) is 0.620 (95% CI: 0.598–0.641, P < 0.001), indicating modest discriminatory ability. Area under the ROC curve; CAD, coronary artery disease; NEFAs, non-esterified fatty acids; ROC, receiver operating characteristic.

Multivariate analysis, adjusting for various risk factors, showed that NEFAs remained independently associated with ACS risk (OR = 1.207 per 100 μmol/L, 95% CI = 1.163–1.253) (**Table 4**). Restricted cubic spline analysis confirmed a linear relationship between NEFAs and ACS risk (P for nonlinear = 0.494) (**Figure 2**). ROC analysis indicated that NEFAs had significant diagnostic value for ACS, with an AUC of 0.620 (95% CI = 0.598-0.641, *P* < 0.001) (**Figure 3**).

**Table 4:** Risk factors for ACS in young patients in multivariate logistic regression analysis.

Variables	OR	95% CI	P-value
Sex	6.978	5.379-9.058	<0.001
Smoking	1.603	1.256-2.044	<0.001
CAD family history (yes *vs* no)	1.100	0.661-1.831	0.713
DM (yes *vs* no)	1.173	0.930-1.478	0.177
Hypertension (yes *vs* no)	1.589	1.284-1.967	<0.001
TG (per 1 mmol/L)	1.172	1.076-1.277	<0.001
TC (per 1 mmol/L)	0.931	0.793-1.093	0.383
LDL-C (per 1 mmol/L)	1.302	1.060-1.600	0.012
HDL-C (per 1 mmol/L)	1.011	0.658-1.553	0.962
eGFR (per 10 mL/min/1.73 m^2^)	0.875	0.834-0.918	<0.001
hs-CRP (≥3.0 mg/ml *vs <*3.0 mg/ml)	0.997	0.982-1.012	0.706
NEFAs (per 100μmol/L)	1.207	1.163- 1.253	<0.001

Variables with a P <0.10 in univariate logistic regression analysis (listed in **Table 3**) were included in the multivariate logistic regression analysis, using an enter method.

CAD, coronary artery disease; CI, confidence interval; eGFR, estimated glomerular filtration rate; HDL-C, high-density lipoprotein cholesterol; hs-CRP, high-sensitivity C-reactive protein; LDL-C, low-density lipoprotein cholesterol; OR, odds ratio; NEFA, non-esterified fatty acids; TG, triglyceride.

## 4 Discussion

This study uncovered two key findings. First, higher circulating NEFA levels correlated with dyslipidemia (elevated TGs and LDL-C) and increased hs-CRP, an inflammation marker. Second, elevated NEFA levels were linked to a higher ACS risk in young Chinese patients, independent of traditional cardiovascular risk factors like sex, smoking, dyslipidemia, and hs-CRP levels.

Our results align with prior studies linking elevated serum NEFA levels to increased CVD risk. For example, Kan et al. demonstrated in a cross-sectional study of Chinese patients undergoing CAG that elevated NEFA levels serve as an independent risk factor for acute myocardial infarction (19). Similarly, Zhang et al. found that higher NEFA levels were associated with both the presence and severity of CAD and carotid atherosclerotic plaque in patients with type 2 DM, suggesting that NEFAs could be a valuable biomarker for managing diabetic patients (20). In contrast, the Multi-Ethnic Study of Atherosclerosis (MESA) did not find an association between NEFA levels and coronary heart disease (CHD) or CVD incidence (21). Additionally, another study reported a negative association between NEFAs and ischemic cardiomyopathy in heart failure patients with DM (22). These inconsistencies may stem from variations in study populations or methodologies. Our cohort was exclusively Chinese, whereas MESA included multi-ethnic participants. Asians exhibit higher NEFA-driven insulin resistance risk at lower BMI thresholds. Furthermore, the MESA assessed long-term CHD incidence in asymptomatic adults, while we evaluated ACS in symptomatic young patients, likely capturing acute metabolic perturbations. To our knowledge, this study is the first to identify NEFAs as an independent biomarker for premature CVD, specifically in a young population.

The mechanisms linking NEFAs to ACS are still under investigation, with several potential pathways suggested. Firstly, metabolic dysregulation: elevated levels of circulating NEFAs are known to induce insulin resistance and systemic metabolic dysfunction, including dyslipidemia and DM (7, 23). These metabolic disturbances are well-established risk factors for ACS. Our study observed a positive correlation between NEFAs and levels of LDL-C and TGs. Nevertheless, the association between NEFAs and ACS remained significant even after adjusting for these metabolic factors, suggesting that additional mechanisms might be involved. Secondly, endothelial dysfunction: high concentrations of circulating NEFAs may reduce the activity of endothelial nitric oxide synthase, impairing endothelium-dependent vasodilation and leading to endothelial dysfunction (24, 25). Endothelial dysfunction is a recognized risk factor for thrombosis and ACS. Thirdly, inflammatory activation: NEFAs may contribute to ACS through the activation of reactive oxygen species, the sympathetic nervous system, vascular adhesion molecules-1, and chronic inflammation (23, 26, 27). Notably, our study found a positive correlation between NEFAs and hs-CRP, an inflammatory marker, supporting the hypothesis that inflammation may play a significant role in the relationship between NEFAs and atherosclerosis.

In addition to their association with the risk of CVD, circulating NEFAs may also influence the prognosis of patients with CVD. For example, a *post hoc* analysis of the AleCardio trial, which investigated the effect of aleglitazar on CV outcomes in patients with type 2 DM, found that baseline NEFAs levels—rather than changes in NEFAs—were directly associated with an increased risk of major adverse CV events and mortality (28). Similarly, a study of 5,443 Chinese patients reported that baseline NEFA levels were linked to diabetes and pre-diabetes and CAD outcomes, revealing NEFAs as a useful prognostic marker for those with diabetes (29). However, it is unclear if these findings apply to young patients, with or without diabetes.

Given NEFAs’ role in CVD incidence and outcomes, targeted strategies to modulate NEFA levels could enhance primary and secondary prevention. Potential management strategies include lifestyle interventions such as body weight control, regular physical activity, adequate sleep, and smoking cessation (7). Future research should focus on drug development and target identification for modulating circulating NEFAs.

This study has several limitations. First, the case-control design limits causal inference, and reverse causality cannot be ruled out. The sample size may also be inadequate to adjust for numerous confounders. Prospective cohort studies or Mendelian randomization focused on genetic variants related to NEFAs could better establish causality between NEFAs and ACS in young patients. Specifically, Mendelian randomization could utilize genetic variants strongly associated with circulating NEFA levels as instrumental variables, thereby leveraging population-scale genomic datasets to mitigate confounding and establish causal relationships between NEFAs and ACS in young populations. Second, only total NEFA levels were measured, without examining individual NEFAs, which may have different impacts on cardiovascular risk. For example, lauric acid has been negatively associated with coronary heart disease mortality, while dihomo-γ-linolenic acid is positively associated. Additionally, omega-3 fatty acids, especially eicosapentaenoic acid, might protect against early CAD. Future research should explore the effects of individual NEFAs on ACS risk. Third, many controls were categorized based on negative coronary CTA results instead of CAG. Although coronary CTA has high negative predictive value for ruling out CAD, using CTA alone for control classification may have introduced misclassification bias (30, 31). False-negative CTA results (e.g., due to calcification artifacts or small-vessel disease) could lead to inadvertent inclusion of subclinical CAD cases in the control group, potentially attenuating the observed NEFA-ACS association. Finally, while NEFAs showed statistically significant associations with ACS, their moderate discriminative capacity (AUC = 0.620) underscores the need for future studies to evaluate synergistic effects with other biomarkers or clinical parameters.

## 5 Conclusions

Higher circulating NEFA levels are independently associated with ACS risks in young Chinese adults, potentially mediated by dyslipidemia and inflammation.

## Data Availability

The raw data supporting the conclusions of this article will be made available by the authors, without undue reservation.
